# Correcting inaccurate background mortality in excess hazard models through breakpoints

**DOI:** 10.1186/s12874-020-01139-z

**Published:** 2020-10-29

**Authors:** Robert Darlin Mba, Juste Aristide Goungounga, Nathalie Grafféo, Roch Giorgi, CENSUR working survival group

**Affiliations:** 1 Aix Marseille Univ, Inserm, IRD, SESSTIM, Sciences Économiques & Sociales de la Santé & Traitement de l’Information Médicale, 27 Boulevard Jean Moulin, 13005 Marseille, France; 2 Institut Paoli-Calmettes, Département de la Recherche Clinique et de l’innovation, Marseille, France; 3 Aix Marseille Univ, APHM, Inserm, IRD, SESSTIM, Hop Timone, BioSTIC, Marseille, France

**Keywords:** Excess mortality, Background mortality, Net survival, Additional variable, Breakpoint, Life table, Cancer

## Abstract

**Background:**

Methods for estimating relative survival are widely used in population-based cancer survival studies. These methods are based on splitting the observed (the overall) mortality into excess mortality (due to cancer) and background mortality (due to other causes, as expected in the general population). The latter is derived from life tables usually stratified by age, sex, and calendar year but not by other covariates (such as the deprivation level or the socioeconomic status) which may lack though they would influence background mortality. The absence of these covariates leads to inaccurate background mortality, thus to biases in estimating the excess mortality. These biases may be avoided by adjusting the background mortality for these covariates whenever available.

**Methods:**

In this work, we propose a regression model of excess mortality that corrects for potentially inaccurate background mortality by introducing age-dependent multiplicative parameters through breakpoints, which gives some flexibility. The performance of this model was first assessed with a single and two breakpoints in an intensive simulation study, then the method was applied to French population-based data on colorectal cancer.

**Results:**

The proposed model proved to be interesting in the simulations and the applications to real data; it limited the bias in parameter estimates of the excess mortality in several scenarios and improved the results and the generalizability of Touraine’s proportional hazards model.

**Conclusion:**

Finally, the proposed model is a good approach to correct reliably inaccurate background mortality by introducing multiplicative parameters that depend on age and on an additional variable through breakpoints.

## Background

Many medical research works dedicated to prognosis or to the impact of some covariates on a given disease outcome rely largely on population-based indicators. In cancer epidemiology, using observational data from cancer registry, survival after cancer diagnosis is the most widely used indicator but there are now several aspects of survival. Among these aspects, net survival is especially interesting because it provides the survival that would be observed if only deaths from cancer were considered [1]; it eliminates the part of mortality due to other causes and allows then fair comparisons between populations or periods [2]. Unfortunately, in cancer registries, the causes of death are often unreliable [3, 4]. For this purpose, methods for estimating excess mortality that do not rely on the cause of death have been developed.

These methods may be applied in a parametric framework [5–8] or a non-parametric framework [9–12]. In the parametric framework, the models consist in splitting *λ*_*O*_, the observed (or overall) mortality, into two components: *λ*_*E*_, the excess mortality due to the disease, and *λ*_*P*_, the background mortality (i.e., the expected mortality due to other causes in the general population). The latter component is usually derived from life tables adjusted for age, sex, and calendar year. More formally,

$$ {\lambda}_O\left(t|z\right)={\lambda}_E\left(t|z\right)+{\lambda}_P\left(a+t|{z}_D\right). $$

*a* represents the age at diagnosis, *t* the time since diagnosis, and *z* a vector of covariates that includes vector *z*_*D*_. The availability of covariates *z*_*D*_ varies between countries and some are not available at the population level; this may affect the estimates of both the excess mortality and the background mortality. For instance, both cancer-specific mortality and all-cause mortality may differ according to the socioeconomic status [13, 14] and, in some populations, the deprivation level is associated with reduced life expectancy [15]. Moreover, it has been shown that overlooking certain relevant covariates in estimating the background mortality induces a bias in estimating the effects of these covariates on the excess mortality [16, 17]. Thus, searching for more accurate estimates of excess mortality should take into account differences in background mortality due to specific covariates.

When population data are not available, several approaches have been proposed to overcome the problem of insufficiently stratified life tables using individual information. For instance, in a Bayesian framework, Morfeld and McCunney [18] proposed standardized mortality ratios (SMRs) using some prior distributions. Other authors proposed methods to construct life tables stratified by additional variables such as ethnicity [19, 20], socioeconomic deprivation [16, 21, 22], or smoking status [23]. Bower et al. [24] proposed adjusting the background mortality for covariates other than age, sex, and calendar year using information from a control population that would accurately match the reference population. Such approaches allow to improve the estimate of the excess mortality by correcting the background mortality through involving a specific variable, which may be of particular interest in some epidemiological studies. However, although it is best to use external information, on the whole or on a part of the reference population, to construct life tables stratified by additional variables, it is important to note that this is not always possible. Indeed, such external information do not exist and/or are not available at regional or national level. An alternative approach focusing on correction involving a specific variable is based on modelling. Within the context of long-term clinical trials of cancer treatment, Cheuvart and Ryan [25] proposed a model for rescaling patients’ background mortality using a single multiplicative parameter. However, this model relied on aggregate data, which may have involved a loss of information. Furthermore, this scale parameter was common to all patients and allowed the mortality from other causes to differ between the studied group and the general population. Therefore, Touraine et al. [26] proposed a model where the population hazard is modelled using life table mortality rates and multiplicative parameters that depend on the level of an additional variable. However, this model relies on an assumption of proportional hazards; i.e., the background mortality differs from the life table mortality in a multiplicative way. This assumption (which cannot always be checked) may not be true with certain covariates or at certain age intervals. For instance, in the American life tables (that include ethnicity), the background mortality functions of Blacks and Whites deviate from proportionality and intersect between ages 80 and 90.

In line with the modelling approach of correcting a life table by adjusting the population hazard for an additional variable, the aim of the present study was to relax the assumption of proportional hazards between the levels of the additional variable. To this end, the study proposes a model with age-dependent multiplicative parameters using breakpoints. This allows the effect of the additional variable on the background mortality to change according to age.

The present manuscript is organized as follows: the Methods section presents Estève’s model --considered as the classical model for estimating the effects of certain covariates on the excess mortality--, Touraine’s model, the proposed model, and the simulation study. The Results section presents the results of the assessment of the empirical performance of each model through intensive simulations. This section also illustrates the use of these methods on French population-based colorectal cancer data. The manuscript ends with practical recommendations, a discussion about the study limitations, and ways for further research.

## Methods

### Estève’s model (Model 1)

The model proposed by Estève et al. [5] assumes that, at time *t* after diagnosis of a subject aged *a* at diagnosis and considering a vector of covariates *z* that includes a vector of demographic variables *z*_*D*_, the observed hazard of death *λ*_*O*_ may be written:

1 $$ {\lambda}_O\left(t|z\right)={\lambda}_E\left(t|z\right)+{\lambda}_P^{\ast}\left(a+t|{z}_D\right) $$

The first component, *λ*_*E*_, is the excess hazard. It represents the disease-related mortality function and may be expressed as:

$$ {\lambda}_E\left(t|z\right)={\sum}_{k=1}^K\exp \left({\tau}_k\right){I}_k(t)\exp \left({\beta}^Tz\right) $$

In this expression, *β*^*T*^ is the transpose matrix of vector *β* (the latter being the vector of regression parameters which are the logarithms of the hazard ratios), *τ*_*k*_ is the logarithm of the baseline excess hazard in the *k* ^th^ interval for a subject with *z* = 0, *I*_*k*_ is an indicator function (*I*_*k*_ =1 when *t*_*k* − 1_ < *t* < *t*_*k*_, 0 otherwise. These time points are predefined if there is an a priori epidemiological knowledge or they are chosen according to the proportion of deaths in each interval). The baseline excess hazard is a piecewise constant over *K* intervals. The vector of regression parameters *β* and the baseline parameters *τ*_*k*_ (*k* = 1, …, *K*) are estimated by the maximum likelihood method.

The second component, ${\lambda}_P^{\ast }$, is the background mortality. This population-hazard term is not estimated from the data but derived from a life table adjusted for some common variables such as age, sex, and calendar year. These variables belong to *z*_*D*_, which is a subset of *z*.

### Touraine’s model (Model 2)

Touraine et al. [26] proposed a model that allows the background mortality of the studied population to differ from that of the general population using a multiplicative parameter. Unlike Estève’s model, Touraine’s model takes into account an additional variable by which the life table is not initially stratified. This parameter that multiplies the potentially inaccurate background mortality represents the effect of the additional variable on the background mortality. There may exist as many multiplicative parameters as levels of the additional variable. More formally, considering a categorical variable *x* with *M* levels (included in *z*) and a life table not stratified by *x* (i.e., vector of variables *z*_*D*_ does not include *x*), Touraine’s model may be written:

Model  2 $$ {\lambda}_O\left(t|z\right)={\lambda}_E\left(t|z\right)+{\sum}_m{\alpha}_mI\left(x=m\right){\lambda}_P^{\ast}\left(a+t|{z}_D\right)\ \mathrm{with}\ m= 1,\dots, M $$

In this expression, *λ*_*E*_ and ${\lambda}_P^{\ast }$ are defined as in Model 1, *I* is an indicator function (*I* =1 when *x* = *m*, 0 otherwise), and *α*_*m*_ are multiplicative parameters that correct a potentially inaccurate background mortality of subjects whose additional variable *x* = *m*. As in Model 1, parameters *β* and *τ*_*k*_ of the excess mortality and *α*_*m*_ are simultaneously estimated by the maximum likelihood method, ${\overset{\sim }{\alpha}}_m$ is estimated so that ${e}^{{\overset{\sim }{\alpha}}_m}={\alpha}_m$. Similarly, ${\lambda}_P^{\ast }$ is not estimated from the data but derived from a life table.

### Proposed model (Model 3)

The proposed model is an extension of Touraine’s model; it allows the background mortality of the studied population to differ from that of the general population by introducing an age-dependent multiplicative parameter through breakpoints. This means that the effect of the additional variable on the background mortality could be not constant over time and that there may not be constant proportionality between the background mortality functions associated with the levels of the additional variable.

#### The model with B breakpoints

As in Model 2, let us consider *x* with *M* levels and a vector *Ɛ* (*ε*_1_ < *ε*_2_ < … < *ε*_*B*_) of *B* breakpoints. The proposed model may be written:

Model  3 $$ {\lambda}_O\left(t|z\right)={\lambda}_E\left(t|z\right)+{\sum}_m{\sum}_b{\alpha}_{mb}{I}_b\left(a+t|x=m\right){\lambda}_P^{\ast}\left(a+t|{z}_D\right) $$

with *m = 1, …, M*; *b = 1, …, B + 1*. In this equation, *λ*_*E*_ and ${\lambda}_P^{\ast }$ are defined as in Models 1 and 2, *α*_*mb*_ are multiplicative parameters that correct a potentially inaccurate background mortality of subjects whose additional variable *x* = *m* over segment *b*, and *I*_*b*_ is an indicator function (*I*_*b*_ =1 when *ε*_*b* − 1_ ≤ *a* + *t* < *ε*_*b*_, 0 otherwise). As in Models 1 and 2, parameters *β* and *τ*_*k*_ of the excess mortality and *α*_*mb*_ (*m = 1, …, M; b = 1, …, B + 1*) are simultaneously estimated by the maximum likelihood method, ${\overset{\sim }{\alpha}}_{mb}$ is estimated so that ${e}^{{\overset{\sim }{\alpha}}_{mb}}={\alpha}_{mb}$. Similarly, ${\lambda}_P^{\ast }$ is not estimated from the data but derived from a life table. The log-likelihood is defined as:

$$ l\left(\psi \right)={\sum}_{i=1}^n\left(-{\Lambda}_E\left({t}_i|{z}_i\right)-{\sum}_m{\sum}_b{\alpha}_{mb}{I}_b\left({a}_i+{t}_i|{x}_i=m\right){\Lambda}_P^{\ast}\left({a}_i+{t}_i|{z}_{D_i}\right)+{\delta}_i\mathit{\log}\left[{\lambda}_E\left({t}_i|{z}_i\right)+{\sum}_m{\sum}_b{\alpha}_{mb}{I}_b\left({a}_i+{t}_i|{x}_i=m\right){\lambda}_P^{\ast}\left({a}_i+{t}_i|{z}_{D_i}\right)\right]\ \right) $$

where *ψ* = (*β*, *τ*_*k*_, *α*_*mb*_) represents the vector of model parameters, *n* the number of subjects, *δ*_*i*_ the indicator of death for subject *i*, Λ_*E*_ the cumulative excess hazard function, and ${\Lambda}_P^{\ast }$ the cumulative population hazard function. Unlike Model 1, the latter does not cancel when maximizing the log-likelihood. It is easily computed because ${\lambda}_P^{\ast }$ is a piecewise constant function derived from a life table that provides mortality rates by age and calendar year units.

From this log-likelihood, the first derivatives are:

$$ \frac{\partial l\left(\psi \right)}{\partial {\beta}_l}=\sum \limits_{i=1}^n\left(-{z}_{il}{\Lambda}_E\left({t}_i|{z}_i\right)+{\delta}_i\frac{z_{il}{\lambda}_E\left({t}_{\mathrm{i}}|{z}_i\right)}{\lambda_E\left({t}_i|{z}_i\right)+\sum \limits_m\sum \limits_b{\alpha}_{mb}{I}_b\left({a}_i+{t}_i|{x}_i=m\right){\lambda}_P^{\ast}\left({a}_i+{t}_i|{z}_{D_i}\right)}\ \right) $$

$$ \frac{\partial l\left(\psi \right)}{\partial {\tau}_k}=\sum \limits_{i=1}^n\left(-\exp \left({\tau}_k\right){t}_{ki}\exp \left({\beta}^T{z}_i\right)+{\delta}_i\frac{\exp \left({\tau}_k\right){I}_k\left({t}_i\right)\exp \left({\beta}^T{z}_i\right)}{\lambda_E\left({t}_i|{z}_i\right)+\sum \limits_m\sum \limits_b{\alpha}_{mb}{I}_b\left({a}_i+{t}_i|{x}_i=m\right){\lambda}_P^{\ast}\left({a}_i+{t}_i|{z}_{D_i}\right)}\right) $$

$$ \frac{\partial l\left(\psi \right)}{\partial {\overset{\sim }{\alpha}}_{mb}}=\sum \limits_{i=1}^n\left(-{\alpha}_{mb}{I}_b\left({a}_i+{t}_i|{x}_i=m\right){\Lambda}_P^{\ast}\left({a}_i+{t}_i|{z}_{D_i}\right)+{\delta}_i\frac{\alpha_{mb}{I}_b\left({a}_i+{t}_i|{x}_i=m\right){\lambda}_P^{\ast}\left({a}_i+{t}_i|{z}_{D_i}\right)}{\lambda_E\left({t}_i|{z}_i\right)+\sum \limits_m\sum \limits_b{\alpha}_{mb}{I}_b\left({a}_i+{t}_i|{x}_i=m\right){\lambda}_P^{\ast}\left({a}_i+{t}_i|{z}_{D_i}\right)}\right) $$

In these derivatives, *z*_*il*_ is the component *l* of the vector of covariates of subject *i* and *t*_*ki*_ is the time spent in the *k*^th^ interval by subject *i*.

#### Breakpoint number and location

Model 3 assumes that both the number and the locations of the breakpoints are fixed. The literature has reported several approaches for determining the number and the locations of the breakpoints. In the case of a single breakpoint, Kunst et al. [27] proposed a graphical method to determine the location of the breakpoint by a simple examination of a scatter plot. Some authors developed an exact or grid-search type algorithm for breakpoint determination [28–30]. Others proposed a Bayesian MCMC approach [31, 32] but faced a computational bulk even with simple models. Braun et al. [33] proposed an approach using quasi-deviance to measure the quality of the fitted model and adapted the Schwarz criterion for the choice of the number of breakpoints. Molinari et al. [34] and Bessaoud et al. [35] proposed a heuristic approach. More specifically, the range of the variable of interest is divided into 10 segments (i.e. 9 breakpoints since there are the lower and upper bounds of the variable). Thus, in case of *B* breakpoints, there are $\left(\genfrac{}{}{0pt}{}{9}{B}\right)=\frac{9!}{B!\left(9-B\right)!}$ potential vectors of location. For each combination of potential locations, a Bayesian Information Criterion (BIC) is calculated and used to select the best model (the one with lowest criterion). Muggeo [36] proposed a linearization technique where a single or more breakpoints are parameters of the model. Goodman et al. [37] proposed a sequential process. To find the model with the optimal number of breakpoints k (k = 0, …, K) that best fits the data, they performed sequential testings; i.e., they compared successively model pairs (with k vs. k + 1 breakpoints) until failing to reject the null hypothesis (no breakpoint against the alternative of a single breakpoint), which made them retain k and not k + 1 breakpoints.

In this work, we investigated more specifically models with a single (*ε*) and two breakpoints (*ε*_1_, *ε*_2_). Indeed, we have chosen this limited number of breakpoints since it allows the number of parameters to remain low while ensuring a sufficient flexibility to reflect plausible patterns of changes in background mortality over age. Model 3 with a single (Model 3.1) and two (Model 3.2) breakpoints may then be written, respectively:

Model  3.1 $$ {\lambda}_O\left(t|z\right)={\lambda}_E\left(t|z\right)+{\sum}_m\left[{\alpha}_{m1}\ I\left(a+t\le \varepsilon |x=m\right)+{\alpha}_{m2}\ I\left(a+t>\varepsilon |x=m\right)\right]{\lambda}_P^{\ast}\left(a+t|{z}_D\right) $$

$$ {\lambda}_O\left(t|z\right)={\lambda}_E\left(t|z\right)+{\sum}_m\left[\ {\alpha}_{m1}\ I\left(a+t\le {\varepsilon}_1|x=m\right)+{\alpha}_{m2}\ I\left({\varepsilon}_1<a+t\le {\varepsilon}_2|x=m\right)\right. $$

Model  3.2 $$ \left.+{\alpha}_{m3}\ I\left(a+t>{\varepsilon}_2|x=m\right)\right]{\lambda}_P^{\ast}\left(a+t|{z}_D\right) $$

To determine the location of breakpoint(s), we retained the strategy of Molinari et al. [34] and Bessaoud et al. [35]. Thus, there are 9 potential locations with a single breakpoint and 36 combinations of potential locations with two breakpoints. The one with the lowest Akaike Information Criterion (AIC) is selected.

To estimate the parameters of the proposed model, standard optimization functions from R software were used to maximize the log-likelihood (programs available on request).

### Simulations

Intensive simulations were used to assess the performance of the proposed Model 3 (Models 3.1 and 3.2). We also considered selecting between Models 3.1 and 3.2 using AIC, in order to identify the model favoured by the data, and this model is referred to as Model 4. Comparisons were made with the performance of Estève’s and Touraine’s models.

#### Design

Each simulation considered *N* = 1000 samples of *n* = 2000 subjects. A first life table --considered as ‘incomplete’-- was used to construct a ‘complete’ life table; i.e., a life table adjusted for an additional variable. The complete life table was used to generate *T*_*P*_, the time to death from other causes than cancer (which allows deriving the background mortality), *T*_*E*_, the time to death from cancer (which allows deriving the excess mortality), and a censoring time. These times are assumed to be independent conditionally on *z*. The observed time to death was considered as the lowest value between *T*_*P*_, *T*_*E*_, and the censoring time.

Within each simulation, several scenarios were considered by varying the impact of the additional variable on the background mortality. The models’ parameters were estimated from the incomplete life table and the estimates of the covariate effects on the excess mortality were used to compare model performances. The performance criteria were: the bias, the relative bias, the empirical coverage rate (ECR), and the root mean squared error (RMSE). Model selection used the AIC which allows model penalization according to the number of parameters to satisfy parameter parsimony. The model with the lowest AIC was the best model.

#### Simulated data

##### Patient covariates and excess mortality

The age at cancer diagnosis (*a*) and the additional variable (*x*) were the covariates that influenced the excess mortality. *a* was simulated from a mixture of uniform distributions with 25% of subjects in age class [30–65[, 35% in [65–75[, and 40% in [75–85[. *x* was simulated as a binary variable with occurrence probability *p* = 90% or 10%. The baseline excess hazard was the hazard function of a generalized Weibull distribution [38] with parameters (*k*, *λ*, *θ*) = (2, 0.2, 0.5), where *k* is the shape parameter, *λ* the scale parameter and *θ* the location parameter. *T*_*E*_ was then simulated using the inverse transformation method with covariate effects *β*_***a***_ = 0.3 and *β*_***x***_ = − 0.2. The excess mortality was obtained by: *λ*_*E*_(*t*| *a*, *x*) = ${\lambda}_E^0(t){e}^{\beta_aa+{\beta}_xx}$. An administrative censoring was assumed at 6 years, which resulted 40% of censoring rate in generated data. All subjects were considered to be men diagnosed within the same year.

##### Background mortality

The incomplete life table was the one available from function survexp.us in package survival of R. This table provides the background mortality of the American population by age, sex, and calendar year from 1940 to 2014. For simplicity, the incomplete life table was considered as adjusted for age only and the selected background mortality was that of men in year 1990. As *a* and *x* may also influence the background mortality, the complete life table was stratified by *a* and *x* and *T*_*P*_ was simulated from this complete life table. Various mismatches in the life table were considered by varying the impact of ***x*** on the background mortality. This led to six scenarios:

- *Scenario A*: No mismatch; i.e., *x* has no effect on the background mortality.
- *Scenario B*: Proportional mismatch; i.e., the two levels of *x* have proportional effects on the background mortality.
- *Scenario C*: Non-proportional crossover mismatch; i.e., the two levels of *x* have non-proportional effects on the background mortality and the background mortality functions intersect.
- *Scenario D*: Non-proportional converging mismatch; i.e., the two levels of *x* have non-proportional effects on the background mortality and the background mortality functions converge.
- *Scenario E*: Non-proportional diverging mismatch; i.e., the two levels of *x* have non-proportional effects on the background mortality and the background mortality functions diverge.
- *Scenario F*: Non-proportional three-level mismatch; i.e., the three levels of *x* have non-proportional effects on the background mortality.

**Fig. 1:**
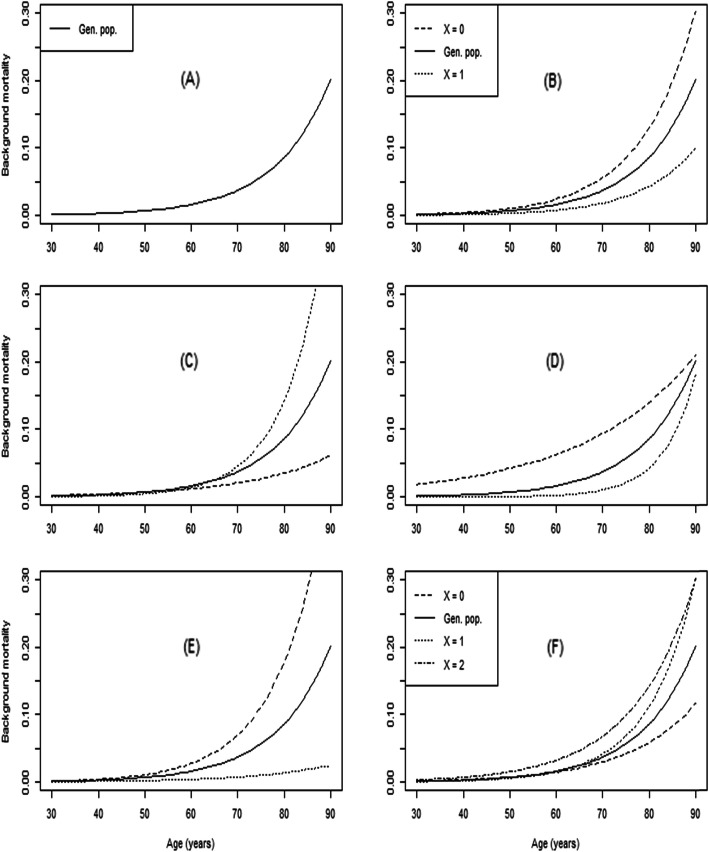
Mismatches (Scenarios A to F) in the life table used for simulation. Note: The solid lines represent the background mortality functions from the incomplete life table. The dotted or dashed lines represent the background mortality functions from the complete life table adjusted for ***x***

All models were run with each of these scenarios. Scenario A ensures that Models 3.1 and 3.2 perform well in the absence of an additional variable. Scenario B ensures that Models 3.1 and 3.2 perform well in the situation taken into account by Model 2. The other scenarios ensure that Models 3.1 and 3.2 improve the performance of Model 2 in some realistic cases. Figure 1 illustrates all these scenarios. It shows, in each scenario, the background mortality according to the incomplete life table (solid line) and the background mortalities associated with various levels of the additional variable of the complete life table (dotted or dashed lines). For a much clearer view, an additional figure shows the mismatches in the life table used for the simulations in patients under 65 years (see Additional file 1).

## Results

### Simulation results

Table 1 displays the bias, the relative bias, the ECR, and the RMSE for ***a*** and ***x*** from all models with Scenarios A to E when the proportion of subjects with ***x*** = 1 is 90% (for proportion = 10%, see Additional file 2). The results from Scenario F when the proportions of subjects with *x* = 1 and *x* = 2 are respectively 10 and 80% are presented in Additional file 3.

**Table 1:** Performance criteria stemming from the simulation study with Scenarios A to E

Scenario	Model	*β*_*a*_ = 0.3				*β*_*x*_ = − 0.2			
		Bias	Rel. bias	ECR	RMSE	Bias	Rel. bias	ECR	RMSE
A	1	−0.012	− 0.039	95.1	0.05	0.002	− 0.012	95.1	0.13
	2	0.006	0.019	94.0	0.08	0.029	−0.146	96.4	0.21
	3.1	−0.001	−0.003	91.4	0.09	0.064	−0.322	96.9	0.25
	3.2	−0.003	−0.009	90.4	0.09	0.077	−0.386	96.6	0.27
	4	0.000	−0.002	91.1	0.09	0.070	−0.348	96.5	0.26
B	1	−0.111	−0.370	30.1	0.12	−0.366	1.832	18.2	0.39
	2	0.008	0.026	90.5	0.08	0.010	−0.050	95.6	0.23
	3.1	0.001	0.003	90.6	0.08	0.040	−0.201	94.9	0.27
	3.2	0.000	−0.001	91.4	0.08	0.050	−0.252	94.7	0.29
	4	0.000	0.000	91.4	0.08	0.045	−0.224	94.6	0.28
C	1	0.135	0.450	14.6	0.14	0.388	−1.940	17.7	0.41
	2	−0.068	−0.227	88.5	0.11	−0.181	0.904	82.0	0.27
	3.1	−0.049	−0.163	90.9	0.10	−0.068	0.338	93.3	0.25
	3.2	− 0.051	− 0.171	91.7	0.10	−0.051	0.256	94.1	0.25
	4	−0.049	− 0.163	91.2	0.10	−0.063	0.316	93.6	0.25
D	1	−0.102	−0.340	40.1	0.11	−0.536	2.681	00.6	0.55
	2	−0.052	−0.174	89.2	0.09	−0.329	1.644	57.9	0.39
	3.1	−0.037	−0.122	91.7	0.09	−0.195	0.974	76.7	0.32
	3.2	−0.036	−0.120	91.4	0.08	−0.172	0.860	79.2	0.32
	4	−0.037	− 0.122	91.7	0.09	−0.185	0.927	77.7	0.32
E	1	−0.212	−0.707	00.7	0.22	−0.694	3.468	00.1	0.70
	2	−0.006	−0.019	94.7	0.05	−0.061	0.307	92.3	0.24
	3.1	−0.015	−0.051	94.6	0.06	−0.001	0.004	93.7	0.29
	3.2	−0.020	−0.067	95.3	0.06	0.017	−0.087	94.3	0.30
	4	−0.016	−0.052	94.7	0.06	0.003	−0.013	94.0	0.29

Scenarios A to E: Proportion of subjects with *x* = 1 is 90%

Table 2 displays the percentage of times each model was retained (%AIC) with Scenarios A to E when the proportion of subjects with ***x*** = 1 is 90% (for proportion = 10%, see Additional file 4) and Scenario F.

**Table 2:** Percentage of times each model was retained on the basis of its AIC

Scenario	Model	%AIC	Model	%AIC	Model	%AIC
A	1	66.70	1	66.70	1	64.78
	2	08.40	2	13.46	2	08.40
	3.1	24.90	3.2	12.55	4	26.82 (90.89,09.11)^a^
B	1	04.50	1	05.82	1	04.29
	2	44.94	2	67.31	2	43.62
	3.1	50.56	3.2	26.87	4	52.09 (89.58,10.42)^a^
C	1	00.60	1	01.30	1	00.60
	2	25.03	2	51.65	2	24.02
	3.1	74.37	3.2	47.05	4	75.38 (87.49,12.51)^a^
D	1	24.33	1	33.63	1	23.82
	2	12.31	2	23.32	2	12.21
	3.1	63.36	3.2	43.05	4	63.97 (87.69,12.31)^a^
E	1	00.00	1	00.00	1	00.00
	2	54.96	2	77.58	2	52.81
	3.1	45.04	3.2	22.42	4	47.19 (89.97,10.03)^a^
F	1	13.94	1	17.85	1	13.84
	2	34.50	2	55.47	2	33.70
	3.1	51.56	3.2	26.68	4	52.46 (91.27,08.73)^a^

%AIC: Percentage of times each model (between compared models) was retained according AIC

^a^Percentage of times Models 3.1 and 3.2 were retained

Scenarios A to E: Proportion of subjects with *x* = 1 is 90%; Scenario F: Proportions of subjects with *x* = 1 and *x* = 2 are respectively 10 and 80%

**Fig. 2:**
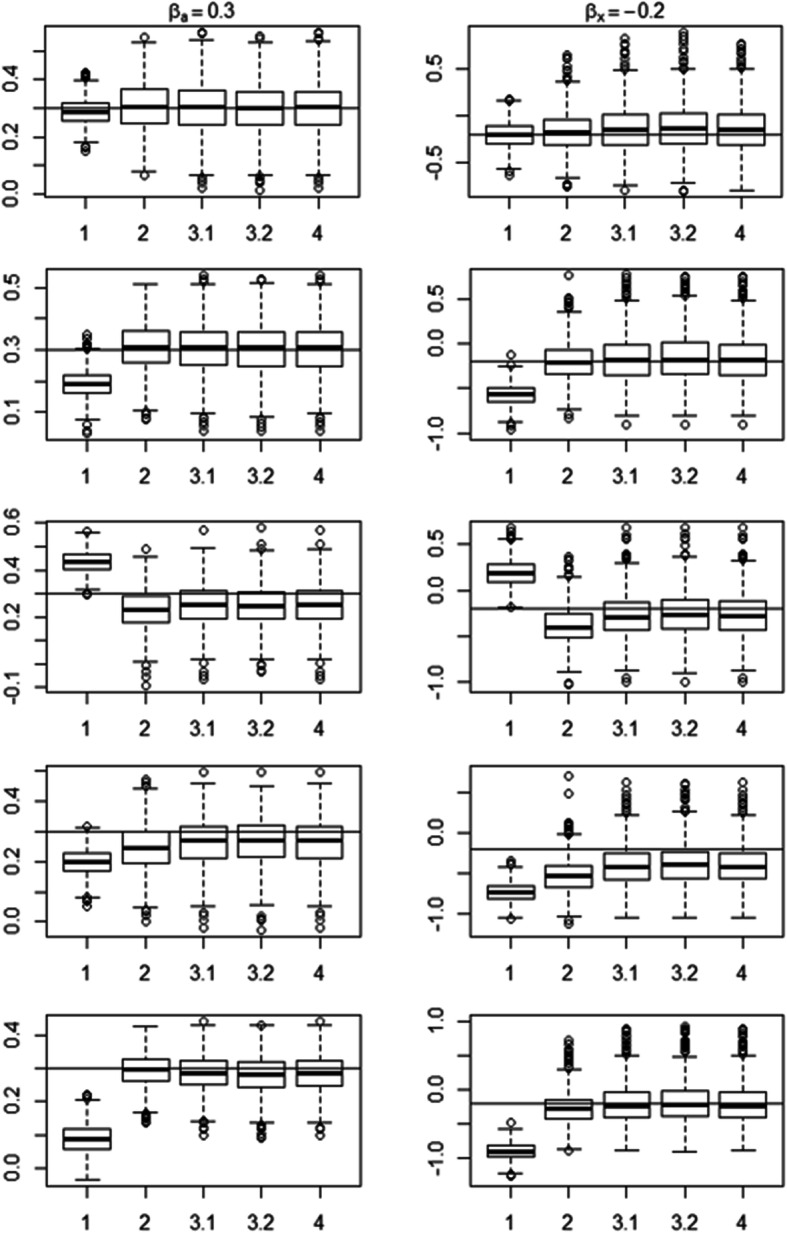
Boxplots of the estimates from the simulation study with Scenarios A to E. Note: Panels from top to bottom correspond to Scenarios A to E, respectively. 1, 2, 3.1, 3.2 and 4 correspond to Models 1, 2, 3.1, 3.2 and 4, respectively. Scenarios A to E: Proportion of subjects with *x* = 1 is 90%

Figure 2 shows the boxplots of the estimates of the effects of covariates ***a*** and ***x*** on the excess mortality in Scenarios A to E when the proportion of subjects with *x* = 1 is 90% (for proportion = 10%, see Additional file 5, and for Scenario F, see Additional file 6). The true values of the parameters lay on the horizontal line.

In Scenario A (No mismatch), the incomplete and complete life table were the same (no use of additional variable); they provided a true value of the background mortality. For *β*_*a*_, Models 3.1 and 3.2 provided unbiased estimates and ECRs equal to 91.4 and 90.4% respectively; for *β*_*x*_, they provided biases close to 0 and ECRs equal to 96.9% and 96.6 respectively. Models 1 and 2 yielded practically the same results. Thus, in the absence of additional variable, Models 3.1 and 3.2 are as performant as Model 1 or 2 although they showed a higher variability vs. Model 1. Model 1 performed better than Models 2, 3.1 and 3.2 in terms of AIC; it was selected nearly seven times out of ten (66.70 and 73.99%). Model 4 was also centered around the true generated values and tended to favour Model 3.1 (90.89%) vs. Model 3.2.

In Scenarios B to F below, the incomplete and complete life tables were different (use of an additional variable). The incomplete life table was used for estimation; it provided inaccurate values of the background mortality.

In Scenario B (Proportional mismatch), for *β*_*a*_, Models 3.1 and 3.2 provided unbiased estimates and ECRs close to 90%; for *β*_*x*_, they provided biases close to 0 and ECRs close to 95%. Model 2 provided the same results. Thus, Models 3.1 and 3.2 performed as well as Model 2 though Model 3.1 was selected only a little more than once in two times (50.56%) while Model 3.2 was selected nearly three times out of ten (26.87%), and Model 4 favoured Model 3.1 (89.58%) vs. Model 3.2. In contrast, Model 1 led to biased parameter estimates, poor ECR, and low AIC-based selection.

In Scenario C (Non-proportional crossover mismatch), Models 3.1 and 3.2 provided biases close to 0 and ECRs greater than 90%; they showed also a lower variability than Models 1 and 2. Model 2 provided biased parameter estimates (of *β*_*x*_ in particular) and ECRs lower than 90%. It underestimated the effect of ***x*** on the excess mortality. Model 1 provided biased parameter estimates, poor ECRs, and low AIC-based selection. It overestimated the effects of the covariates on the excess mortality. As expected, Models 1 and 2 performed poorly while Model 3.1 performed well and was selected nearly three times out of four (74.37%), and Model 4 favoured Model 3.1 vs Model 3.2.

In Scenario D (Non-proportional converging mismatch), for *β*_*a*_, Models 2, 3.1 and 3.2 provided unbiased estimates and ECRs close to 90%, whereas Model 1 provided biased estimates and a poor ECR (40.1%). Models 3.1 and 3.2 provided less biased effects of ***x*** on the excess mortality and ECRs greater than that of Model 2. All models underestimated the effect of ***x*** on the excess mortality. Models 3.1 and 3.2 presented low variability vs. Model 1 or Model 2 and were selected nearly six times out of ten (63.36%) and four times out of ten (43.05%) respectively. Model 4 favoured Model 3.1 vs Model 3.2.

In Scenario E (Non-proportional diverging mismatch), Models 2, 3.1 and 3.2 provided biases close to 0 and ECRs greater than 90%, whereas Model 1 provided biased estimates, ECRs close to 0 and underestimated the effects of the covariates on the excess mortality. Model 3.1 is always favoured over Model 3.2 (89.97% vs. 10.03%). This scenario highlighted significantly the fact that Model 1 does not apply to life tables stratified by an additional variable.

In Scenario F (Non-proportional 3-level mismatch), Models 3.1 and 3.2 provided less biased effects of the covariates than Model 1 or Model 2 and ECRs greater than 90%. Model 1 provided small ECRs and overestimated the effects of the covariates on the excess mortality, whereas the other three models underestimated them. In addition, Model 3.1 was selected about half the times (51.56%) and favoured over Model 3.2. Furthermore, whenever the levels of the additional variable had small effects on the background mortality (not shown here), the four models gave comparable results.

### Applications to population-based data on colorectal cancer

The interest of the proposed model and performance comparison between Models 1 and 2 were tested on population-based data on colorectal cancer from nine French cancer registries of network FRANCIM. This testing analyzed mortality data on 1398 patients with colorectal cancer diagnosed between January 1 and December 31, 1995.

Two applications studied the effects of three prognostic factors on the excess mortality: sex, age at diagnosis, and cancer stage at diagnosis. These applications excluded patients with missing data on any factor and patients aged > 90 years. Age at diagnosis was considered under three categories (≤ 64, 65–74, and 75–90) and cancer stage categories III and IV were merged to balance the number of patients into three categories.

#### Application 1

##### Method

In Application 1, Model 1 was used with the ‘complete’ French life table that included covariates age, sex, and calendar year and was considered as the gold standard (Model 1*). The purpose of this model was to provide reference values as in a simulation study. Models 1, 2, and 3 (3.1 and 3.2) were used with the ‘incomplete’ life table that included covariates age and calendar year only; covariate sex was considered as the additional variable.

##### Results

Table 3 shows the covariates used for the estimation of the excess mortality.

**Table 3:** Covariate categories of the study’s colorectal cancer patients

Covariates	Application 1	Application 2
Cohort size	1304	788
Age		
≤ 64 years	374 (28.7)^a^	273 (34.6)^a^
65–74 years	460 (35.3)	267 (33.9)
75–90 years	470 (36.0)	248 (31.5)
Sex		
Men	704 (54.0)	454 (57.6)
Women	600 (46.0)	334 (42.4)
Cancer stage		
I	391 (30.0)	229 (29.1)
II	486 (37.3)	289 (36.6)
III-IV	427 (32.7)	270 (34.3)
Socioprofessional category		
No occupational activity		119 (15.1)
Clerical and manual workers		340 (43.1)
Farmers		125 (15.9)
Other occupational activities		204 (25.9)

^a^Column percentage

Patients’ ages ranged from 21 to 90 years (mean = 69.8) and category sizes were rather balanced. At 10 years post-diagnosis, 664 deaths had occurred (i.e., 50.3% of 1304 included patients). The results of the application of the models are summarized in Table 4. AIC values for Model 3 with a single and two breakpoints were calculated (4230 and 4228, respectively), resulting in the selection of a model with two-breakpoints, located at 83 and 88 years.

**Table 4:** EHR estimates with data on colorectal cancer using life tables stratified and not stratified by sex

	Model 1*	Model 1	Model 2		Model 3 (Ɛ_1_ = 83, Ɛ_2_ = 88)	
Variables	EHR [95% CI]	EHR [95% CI]	EHR [95% CI]	$\hat{\alpha}$[95% CI]	EHR [95% CI]	$\hat{\alpha}$[95% CI]
Age						
≤ 64	ref.	ref.	ref.		ref.	
65–74	1.37 [1.01–1.87]	1.35 [1.00–1.82]	1.36 [1.00–1.85]		1.37 [1.01–1.87]	
75–90	1.67 [1.21–2.31]	1.69 [1.23–2.33]	1.67 [1.16–2.40]		1.66 [1.15–2.38]	
CS						
I	ref.	ref.	ref.		ref.	
II	5.92 [2.35–14.90]	5.26 [2.32–11.88]	6.47 [1.82–22.95]		5.75 [1.66–19.91]	
III-IV	14.65 [5.91–36.33]	12.57 [5.65–27.99]	16.07 [4.28–60.32]		14.31 [3.85–53.10]	
Sex						
Men	ref.	ref.	ref.	1.33 [0.92–1.92]	ref.	1.42 [0.90–2.24]^a^
						1.24 [0.74–2.07]^b^
						0.66 [0.32–1.39]^c^
Women	0.93 [0.72–1.20]	0.69 [0.53–0.90]	0.91 [0.67–1.24]	0.74 [0.59–0.93]	0.94 [0.70–1.29]	0.62 [0.45–0.84]^a^
						0.77 [0.57–1.02]^b^
						1.74 [0.59–5.11]^c^
AIC	4223	4238	4230		4228	

Note: Model 1* (Gold standard) is estimated using a life table stratified by sex and Models 1, 2, and 3 are estimated using the same life table not stratified by sex. Excess hazard ratio (EHR) with 95% confidence interval (95% CI) are estimated for Models 1*,1,2 and 3, while *α* with 95% CI are estimated for Model 2 and 3. Ɛ_1_, Ɛ_2_, Determined breakpoints; CS, Cancer stage; ^a^ Estimate of *α* before Ɛ_1_; ^b^ Estimate of *α* between Ɛ_1_ and Ɛ_2_, ^c^ Estimate of *α* after Ɛ_2_; AIC, Akaike information criterion

Using the incomplete life table, all three models (Models 1, 2, and 3) found that age and cancer stage at diagnosis were significantly associated with excess mortality. With Model 3, the estimated excess hazard effects (EHR) of these two factors were the closest to the reference values given by Model 1*. With ‘sex’ as an additional variable, Model 1 found wrongly a significant effect (EHR = 0.69 [0.53–0.90]) because this effect was not found with Model 1* (EHR = 0.93 [0.72–1.20]). The estimates of α from Model 2 and Model 3 were broadly consistent with the values obtained with the complete life table because men had a higher and women a lower background mortality than that of the general population from the incomplete life table. Specifically, Model 2 found that, irrespective of age, the background mortalities in men and women were respectively 1.33 and 0.74 times the overall mortality as per the incomplete life table. In contrast, Model 3 showed that the background mortality was different before and after 88 years. Moreover, the AIC values for Model 2 and Model 3 were 4230 and 4228, respectively; and Model 3 was less biased. Model 1 performed the worst in terms of AIC (4238) and had the most biased effects.

#### Application 2

##### Method

In addition to the prognostic factors considered in Application 1, the socioprofessional category (SPC) has also shown an impact on the survival of patients with colorectal cancer [39, 40]. The present application considered four SPCs: no occupational activity, clerical and manual workers, farmers, and other occupational activities.

The use of all models with the available French life table that included covariates age, sex, and calendar year. This life table was not stratified by SPC. Thus, SPC was considered as the additional variable.

##### Results

Patients’ ages ranged from 21 to 90 years (mean = 68.2). Age and stage category sizes were rather balanced; however, clerical or manual workers formed the largest category (Table 3). At 10 years post-diagnosis, 394 deaths had occurred (i.e., 50% of 788 included patients). All models used the inaccurate background mortality from the incomplete life table. The results are summarized in Table 5. The AIC values for Model 3 with single and two breakpoints were the same (2539). We have chosen the model with a single-breakpoint, located at 75 years, which is more parsimonious.

**Table 5:** EHR estimates with data on colorectal cancer using a life table not stratified by SPC

	Model 1	Model 2		Model 3 (Ɛ = 75)	
Variables	EHR [95% CI]	EHR [95% CI]	$\hat{\alpha}$[95% CI]	EHR [95% CI]	$\hat{\alpha}$[95% CI]
Age					
≤ 64	ref.	ref.		ref.	
65–74	1.69 [1.15–2.50]	1.70 [1.16–2.49]		1.74 [1.25–2.43]	
75–90	2.14 [1.43–3.18]	2.21 [1.43–3.42]		2.14 [1.41–3.24]	
Sex					
Men	ref.	ref.		ref.	
Women	0.93 [0.65–1.33]	0.94 [0.63–1.38]		0.80 [0.53–1.21]	
CS					
I	ref.	ref.		ref.	
II	3.71 [1.53–8.99]	2.94 [1.27–6.79]		2.91 [1.21–6.98]	
III-IV	10.96 [4.70–25.53]	8.61 [3.40–21.78]		7.33 [2.48–21.62]	
SPC					
NOA	ref.	ref.	0.72 [0.27–1.90]	ref.	1.54 [0.15–15.96]^a^
					0.53 [0.23–1.24]^b^
CMW	1.23 [0.75–2.03]	1.07 [0.57–2.00]	1.05 [0.59–1.88]	1.31 [0.63–2.70]	0.37 [0.12–1.11]^a^
					1.19 [0.62–2.30]^b^
Farmers	0.72 [0.37–1.39]	0.59 [0.25–1.40]	1.10 [0.63–1.93]	0.85 [0.37–1.94]	0.11 [0.05–0.24]^a^
					1.41 [0.69–2.87]^b^
OOA	1.17 [0.68–2.03]	1.19 [0.61–2.31]	0.70 [0.41–1.20]	1.24 [0.56–2.76]	0.65 [0.23–1.81]^a^
					0.66 [0.38–1.15]^b^
AIC	2539	2545		2539	

Note: Excess hazard ratio (EHR) with 95% confidence interval (95% CI) are estimated for Models 1, 2 and 3, while *α* with 95% CI are estimated for Model 2 and 3. Ɛ, Determined breakpoint; CS, Cancer stage; NOA, No occupational activity; CMW, Clerical and manual workers; OOA, Other occupational activities; ^a^ Estimate of *α* before Ɛ; ^b^ Estimate of *α* after Ɛ; AIC, Akaike information criterion

In this application, all three models (Models 1, 2, and 3) showed that the excess mortality increased significantly with age at diagnosis and cancer stage but no significant difference between men and women. The three models gave close estimates of the effect of age but the effect of cancer stages III-IV (versus I) was greater with Model 1 (EHR = 10.96 [4.70–25.53]) than with Model 2 (EHR = 8.61 [3.40–21.78]) or Model 3 (EHR = 7.33 [2.48–21.62]).

In this application, no model found that additional variable SPC was significantly associated with excess mortality. Model 2 found a lower background mortality in patients with ‘No occupational activity’ and ‘Other occupational activities’ (respectively 0.72 and 0.70 times that provided by the life table) but a higher background mortality in ‘Farmers’ and ‘Clerical and manual workers’ (respectively, 1.10 and 1.05 times that provided by the life table), irrespective of age. In contrast, Model 3 showed that the effect of SPC on the background mortality was not constant over time. Indeed, only patients with “Other occupational activities” had practically the same background mortality before and after 75 years; e.g., ‘Farmers’ had a lower background mortality than the overall mortality from the life table before 75 years but a higher one after that age. In addition, ‘Other occupational activities’ --including intermediate and higher occupations-- had a lower background mortality than the overall mortality from the life table. Furthermore, Model 1 and Model 3 had the same AIC whereas Model 2 performed the worst in terms of AIC (2545).

## Discussion

The present work proposes a regression model of excess mortality (Model 3) able to correct for potentially inaccurate background mortality due to the unavailability of a specific variable on population level. It thus provides an interesting alternative to answer epidemiological questions involving a specific variable affecting both the excess mortality and the background mortality in the absence of life table stratified by this variable and when no external information exists and/or is available to construct such a stratified life table. Specifically, it increases the flexibility of Touraine’s model (Model 2) [26] by introducing age-dependent multiplicative parameters through breakpoints. Whenever a currently available life table is not stratified by an additional variable *x*, Model 3 considers an *x*-specific age-dependent corrective parameter that multiplies the background mortality. In practice, for the proposed Model 3, with a particular focus on model with a single or two breakpoints, we used a heuristic approach to determine the number and locations of breakpoints [34, 35]. We divide age into segments and calculate the AIC for all combinations of *1, 2, ..., B* breakpoints. We retain the one with lowest AIC. As explained above, the choice of this limited number of breakpoints is based on both statistical and epidemiological criteria.

As detailed in the Breakpoint number and location sub-section, other approaches may be used concerning the determination of breakpoint number and location. In addition to graphic and numerical approaches [27–33, 36, 37], it is also possible to “fix” the number and the location based on prior information, for example when a life table stratified by the additional variable *x* exist in another country. More generally, the approaches and strategy are similar to those proposed in the framework of the use of spline functions concerning the choice of the number and location of nodes.

In the simulations, Model 3, whether it’s with a single or two breakpoints, showed a good performance; its estimated parameters and ECRs were close to the nominal values. In all scenario, Model 3 with a single-breakpoint has been favoured over two breakpoints. Thus, Model 3 with a single-breakpoint was sufficient. The simulations also showed that Model 3 was as performant as Model 1 in the absence of additional variable and as performant as Model 2 in case of proportional mismatch. Furthermore, Model 3 eliminated or limited the bias in parameter estimates of the excess mortality in several other mismatch scenarios. However, although it has lower bias, it had a higher variability than Model 2, but was better in terms of AIC. This cost of higher variance may be explained by the additional parameters. Indeed, to estimate the effect of the additional variable on the background mortality, Model 3 has M*B additional multiplicative parameters than Model 2 (*M*: levels of the additional variable; *B*: number of the breakpoints).

In the two practical applications on registry colorectal cancer data and an ‘incomplete’ life table (obtained by removing a variable from a real complete table), Model 3 proved to be useful; it performed better than Models 1 and 2 vs. gold-standard estimates obtained with a ‘complete’ life table. Note that our results differ slightly from those of Touraine et al. work [26] simply because of minor modifications in the choice of criteria for the inclusion of patients in our study. In the second application (SPC as additional variable), Models 1 and 3 had the same AIC. However, the simulations have shown that these models may give comparable results in situations where the effect of the additional variable on the background mortality is not significant (results not shown). Although the 95% CIs of the multiplicative parameters overlap between the two age categories (before and after the breakpoint), there is no interest in determining whether this difference is really significant, or whether there is a way to test for such differences. Indeed, our goal is to correct inaccurate background mortality in excess hazard models in order to eliminate or limit the bias in estimating the effects of prognostic factors on excess mortality.

In addition, the results obtained with Model 3 are consistent with the literature. Indeed, SPCs ‘Farmers’ and ‘Other occupational activities’ have a lower early background mortality (< 65) than the overall mortality from the life table [41]; Farmers would be healthier than the general population [42, 43]. The present study results showed that, before age 75 years, the working population had a lower background mortality than the overall mortality from the general population. People with “No occupational activity” showed a higher background mortality; this may relate to the ‘Healthy Worker Effect’ (healthy individuals keep being employable) [44]. In this work, the socioprofessional category was defined as the longest occupational activity of each subject. Another but highly debatable choice would be the first subject’s occupation.

Given the present results, Model 3 would improve the results of Model 2 by making it more generalizable; specifically, when the assumption of proportionality is not valid at certain age intervals (e.g. in the American life tables that include ethnicity, Black and White background mortality functions deviate from proportionality and intersect between ages 80 and 90). Nevertheless, Model 3 presents some limitations. First, it was found here essentially suitable for estimating the parameters related to a single and necessarily categorical additional variable with no more than two breakpoints. It would be interesting to carry out a study on three or several breakpoints. Nevertheless, the use of several breakpoints may lead to over-parameterization of the model. Fortunately, in medical research, a low number of breakpoints is usually sufficient [34]. Second, Model 3 is still a piecewise proportional population hazards where the parameters related to the additional variable (used to correct the background mortality) vary with age though they remain constant within intervals. Another interesting work would be the use of smooth or flexible functions, such as splines or penalized splines. Then, a generalization to situations where mismatch in the life table may be due to numerous variables may be attractive. A model with a random effect (i.e., a frailty term) was proposed [45]. This term corrects for the effects of several potentially unavailable or unobservable covariates and may differ between subjects, which is of epidemiological interest. Possible limitation of this random effect model, pointed out by the authors, is due to the challenges in estimating the parameters in case of insufficient sample size (simulations done with data sets of size *n = 5000*). Another one may come from the difficulty in interpreting the epidemiological effects captured by the fragility term. However, such model tries to answer to another epidemiological question than the one investigated with our Model 3. In line with multiple mismatches in the life table, an interesting perspective would be to use latent class approach to correct background mortality, which would allow a better description of the epidemiological profiles and their impact on expected mortality.

## Conclusion

In absence of life table stratified by a specific variable and when no external information exists and/or is available to construct life table stratified by this additional variable, the proposed model is a good approach to correct reliably inaccurate background mortality by introducing multiplicative parameters that depend on age and on an additional variable through breakpoints.

## Supplementary information

**Additional file 1.** Mismatches in the life table used for simulations in patients under 65 years old.

**Additional file 2.** Performance criteria stemming from the simulation study with Scenarios A to E.

**Additional file 3.** Performance criteria stemming from the simulation study with Scenario F.

**Additional file 4.** Percentage of times each model was retained on the basis of its AIC.

**Additional file 5.** Boxplots of the estimates from the simulation study with Scenarios A to E.

**Additional file 6.** Boxplots of the estimates from the simulation study with Scenario F.

## Data Availability

The R codes and data used in this paper are available on request from the authors.

## Abbreviations

AIC: Akaike information criterion
BIC: Bayesian information criterion
CI: Confidence interval
CMW: Clerical and manual workers
CS: Cancer stage
ECR: Empirical coverage rate
EHR: Excess hazard ratio
NOA: No occupational activity
OOA: Other occupational activities
RMSE: Root mean squared error
SMR: Standardized mortality ratio
SPC: Socioprofessional category
