# Supplementary figures and images for: Correcting inaccurate background mortality in excess hazard models through breakpoints
Source: BMC Med Res Methodol. 2020 Oct 29;20:268. doi: 10.1186/s12874-020-01139-z (PMC7596976; doi:10.1186/s12874-020-01139-z)

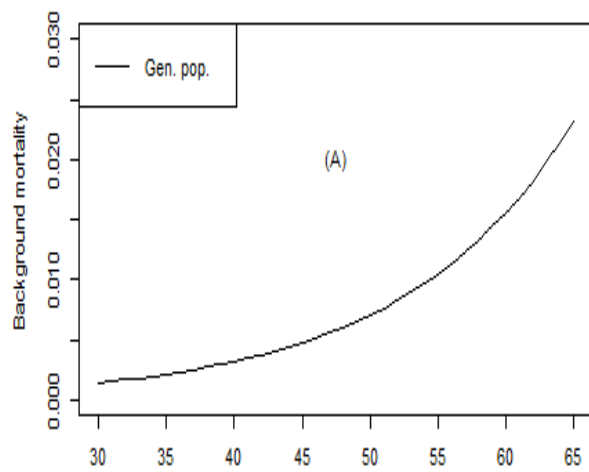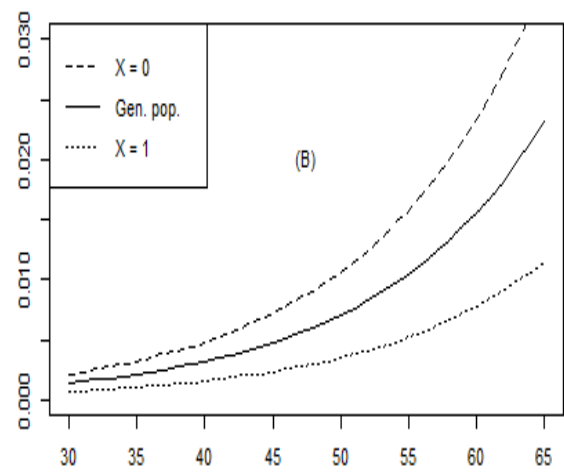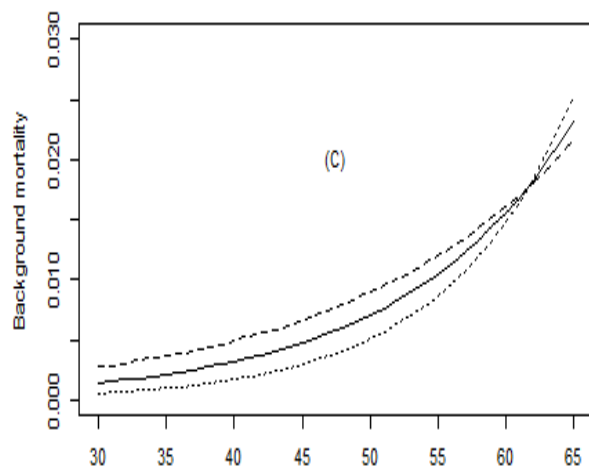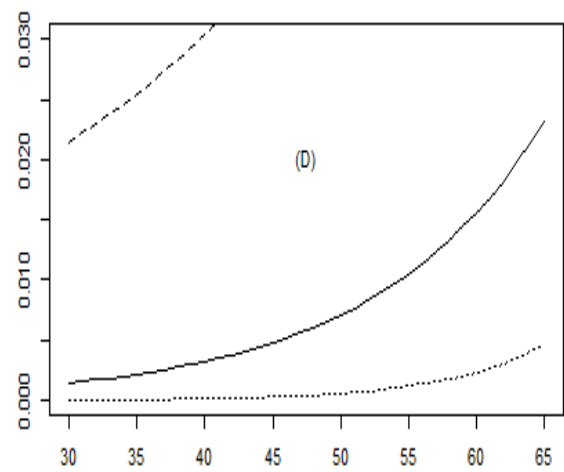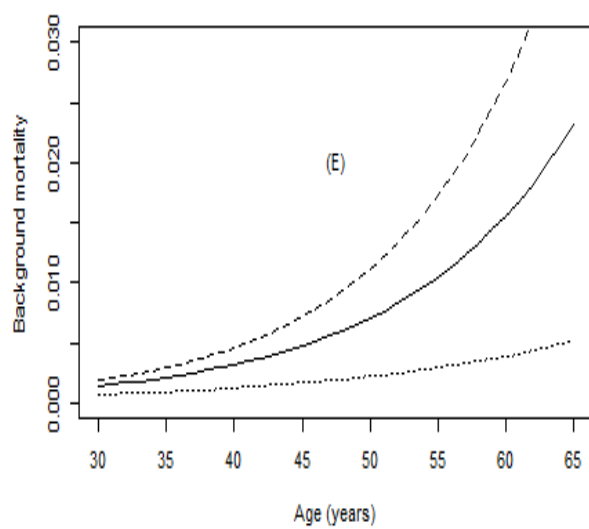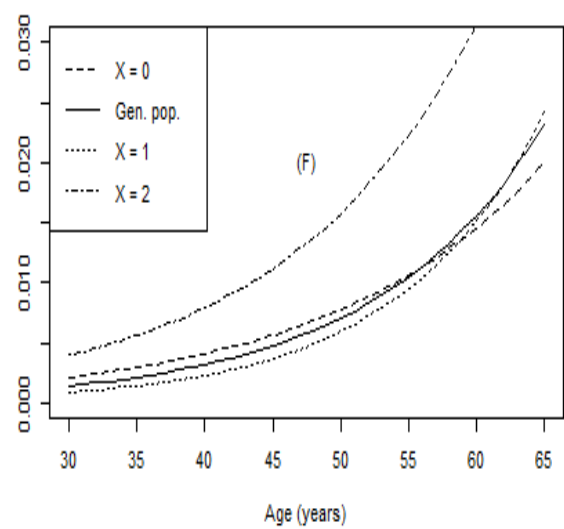

Supplement: Supplementary file 1 — Additional file 1. Mismatches in the life table used for simulations in patients under 65 years old. [file 12874_2020_1139_MOESM1_ESM.pdf]

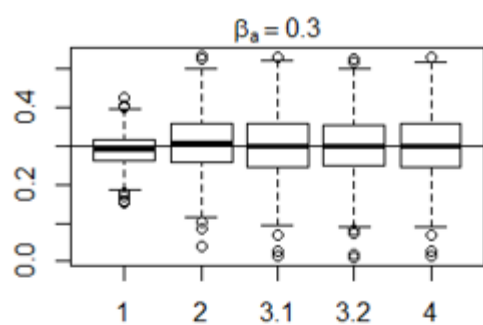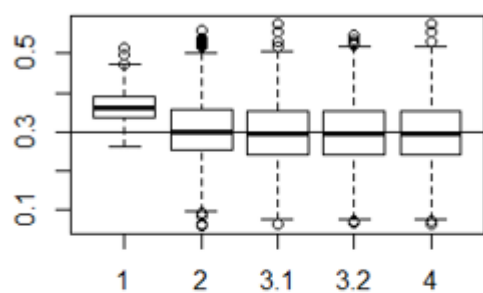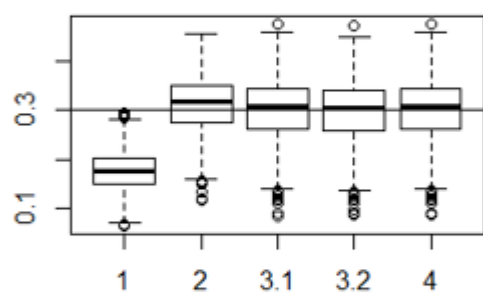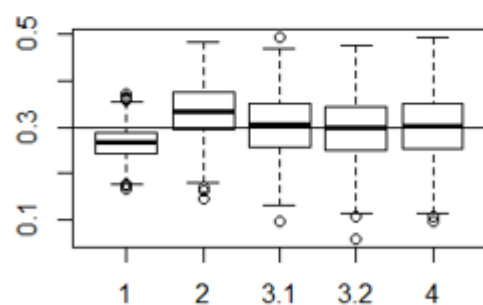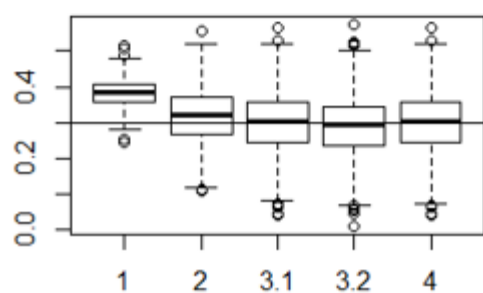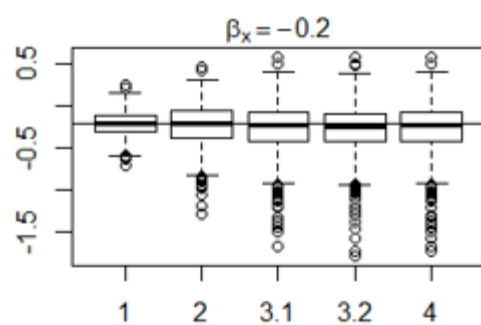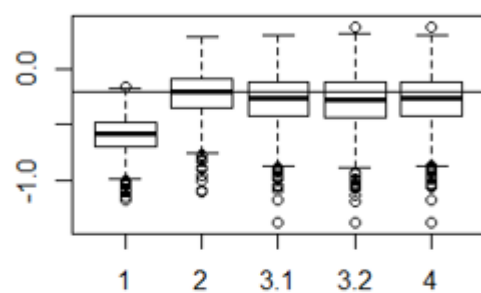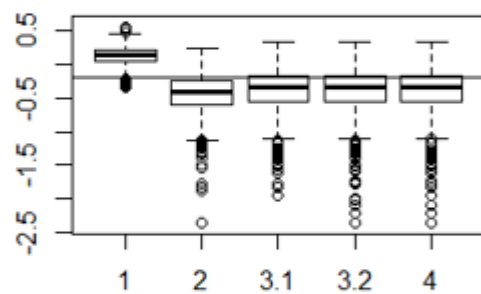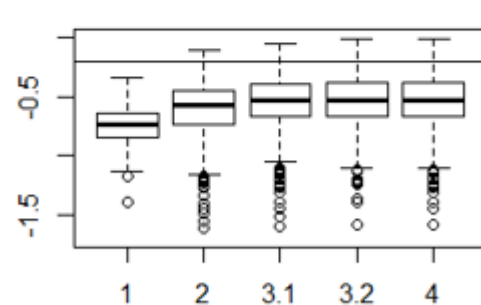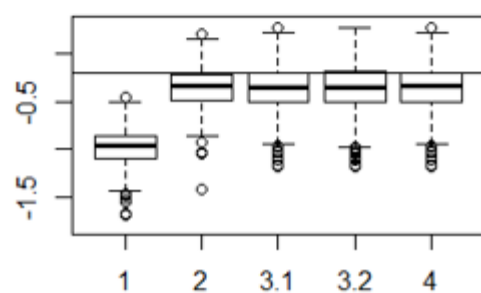

Supplement: Supplementary file 5 — Additional file 5. Boxplots of the estimates from the simulation study with Scenarios A to E. [file 12874_2020_1139_MOESM5_ESM.pdf]

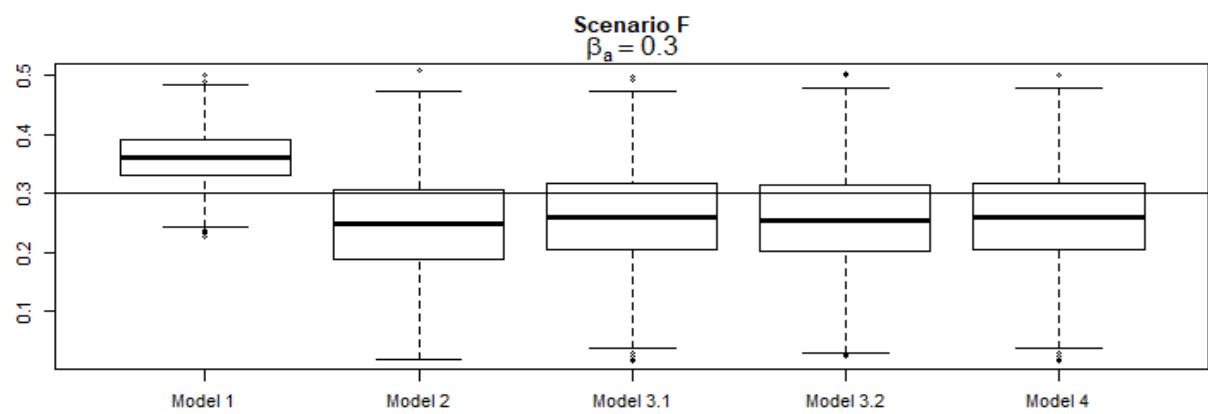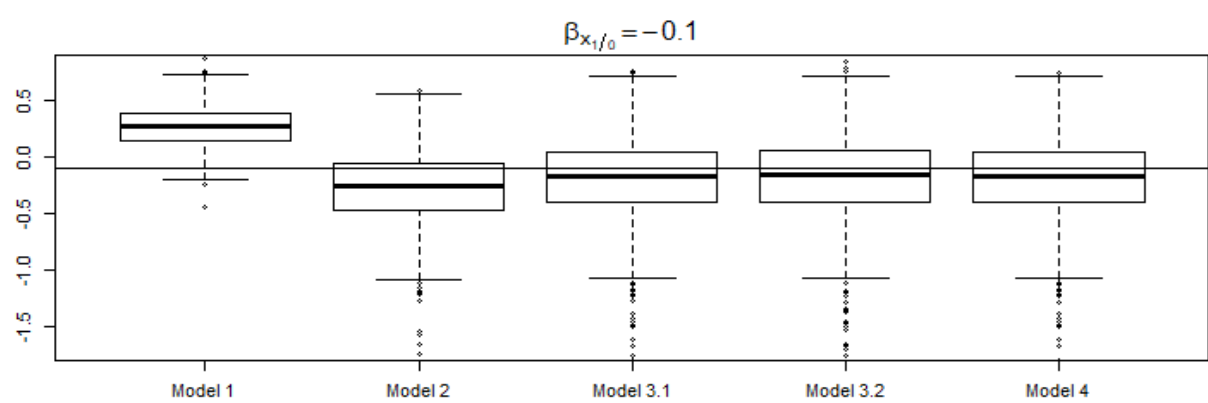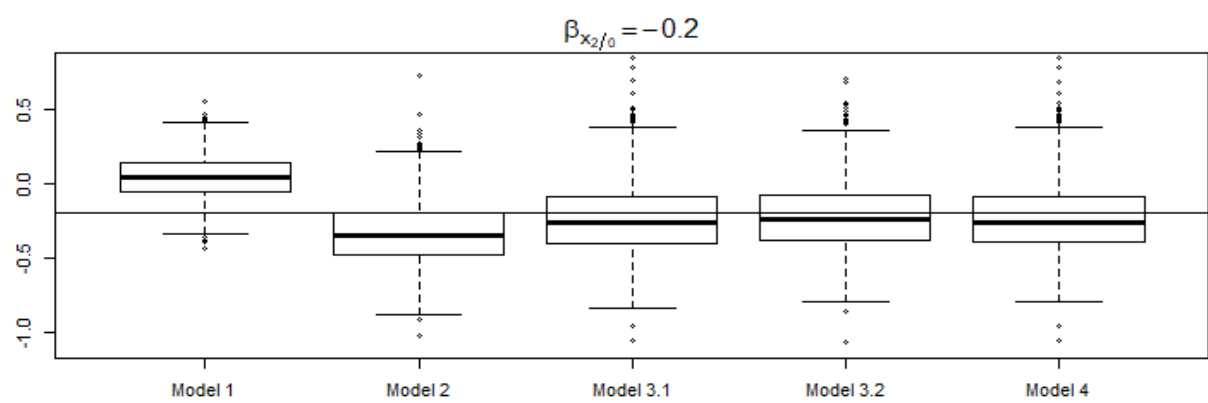

Supplement: Supplementary file 6 — Additional file 6. Boxplots of the estimates from the simulation study with Scenario F. [file 12874_2020_1139_MOESM6_ESM.pdf]
