# Supplementary material for: Correcting inaccurate background mortality in excess hazard models through breakpoints
Source: BMC Med Res Methodol. 2020 Oct 29;20:268. doi: 10.1186/s12874-020-01139-z (PMC7596976; doi:10.1186/s12874-020-01139-z)
Supplement: Supplementary file 2 — Additional file 2. Performance criteria stemming from the simulation study with Scenarios A to E. [file 12874_2020_1139_MOESM2_ESM.pdf]

Scenario	Model	$\beta_a = 0.3$				$\beta_x = -0.2$			
		Bias	Rel. bias	ECR	RMSE	Bias	Rel. bias	ECR	RMSE
A	1	-0.010	-0.034	94.6	0.04	-0.007	0.036	95.2	0.14
	2	0.005	0.018	94.7	0.07	-0.022	0.110	95.7	0.23
	3.1	0.000	0.000	91.9	0.08	-0.064	0.318	95.1	0.29
	3.2	-0.002	-0.005	92.3	0.08	-0.073	0.364	95.6	0.30
	4	-0.001	-0.002	91.9	0.08	-0.068	0.338	95.4	0.30
B	1	0.062	0.207	67.4	0.07	-0.392	1.959	25.5	0.42
	2	0.003	0.012	94.4	0.08	-0.029	0.144	96.4	0.21
	3.1	-0.003	-0.011	93.9	0.08	-0.077	0.383	96.1	0.25
	3.2	-0.007	-0.023	94.3	0.08	-0.091	0.453	96.6	0.27
	4	-0.004	-0.013	94.0	0.08	-0.080	0.402	96.0	0.26
C	1	-0.124	-0.412	13.6	0.13	0.333	-1.664	31.7	0.36
	2	0.010	0.032	90.3	0.06	-0.236	1.180	96.9	0.38
	3.1	0.000	0.000	92.4	0.06	-0.183	0.914	97.2	0.37
	3.2	-0.003	-0.011	93.6	0.06	-0.187	0.935	97.4	0.39
	4	-0.001	-0.002	92.4	0.06	-0.187	0.937	97.1	0.39
D	1	-0.033	-0.109	87.2	0.05	-0.545	2.727	03.0	0.57
	2	0.033	0.109	88.0	0.06	-0.410	2.052	57.2	0.47
	3.1	0.003	0.010	92.8	0.07	-0.351	1.756	71.6	0.42
	3.2	-0.002	-0.007	93.5	0.07	-0.345	1.726	72.5	0.42
	4	0.003	0.010	92.5	0.07	-0.347	1.736	72.1	0.42
E	1	0.084	0.280	41.1	0.09	-0.782	3.910	00.1	0.80
	2	0.019	0.065	94.5	0.07	-0.152	0.759	89.9	0.24
	3.1	0.001	0.003	93.6	0.08	-0.159	0.793	89.2	0.28
	3.2	-0.009	-0.029	93.4	0.08	-0.162	0.811	90.3	0.29
	4	-0.001	-0.004	93.1	0.08	-0.158	0.788	88.9	0.28
