# Supplementary material for: Correcting inaccurate background mortality in excess hazard models through breakpoints
Source: BMC Med Res Methodol. 2020 Oct 29;20:268. doi: 10.1186/s12874-020-01139-z (PMC7596976; doi:10.1186/s12874-020-01139-z)
Supplement: Supplementary file 3 — Additional file 3. Performance criteria stemming from the simulation study with Scenario F. [file 12874_2020_1139_MOESM3_ESM.pdf]

Model	$\beta_a = 0.3$				$\beta_{x_{1/0}} = -0.1$				$\beta_{x_{2/0}} = -0.2$			
	Bias	Rel. bias	ECR	RMSE	Bias	Rel. bias	ECR	RMSE	Bias	Rel. bias	ECR	RMSE
1	0.061	0.204	73.5	0.08	0.357	-3.572	47.1	0.40	0.243	-1.213	61.5	0.28
2	-0.054	-0.179	89.5	0.10	-0.181	1.810	95.8	0.37	-0.137	0.684	84.8	0.25
3.1	-0.041	-0.138	92.8	0.09	-0.107	1.066	96.7	0.39	-0.041	0.207	93.7	0.24
3.2	-0.045	-0.150	92.2	0.10	-0.096	0.958	97.2	0.39	-0.024	0.120	94.0	0.24
4	-0.042	-0.139	92.4	0.09	-0.103	1.035	96.8	0.39	-0.037	0.185	93.8	0.24
