# Supplementary material for: Correcting inaccurate background mortality in excess hazard models through breakpoints
Source: BMC Med Res Methodol. 2020 Oct 29;20:268. doi: 10.1186/s12874-020-01139-z (PMC7596976; doi:10.1186/s12874-020-01139-z)
Supplement: Supplementary file 4 — Additional file 4. Percentage of times each model was retained on the basis of its AIC. [file 12874_2020_1139_MOESM4_ESM.pdf]

Scenario	Model	%AIC	Model	%AIC	Model	%AIC
A	1	66.30	1	74.80	1	64.57
	2	08.00	2	11.94	2	07.69
	3.1	25.71	3.2	13.26	4	27.73 (89.68,10.32)*
B	1	24.72	1	30.15	1	24.42
	2	33.87	2	48.34	2	32.56
	3.1	41.41	3.2	21.51	4	43.02 (89.35,10.65)*
C	1	00.60	1	00.80	1	00.60
	2	46.19	2	70.74	2	44.99
	3.1	53.21	3.2	28.46	4	54.41 (89.78,10.22)*
D	1	40.05	1	47.24	1	38.37
	2	20.86	2	27.94	2	19.42
	3.1	39.09	3.2	24.82	4	42.21 (85.97,14.03)*
E	1	01.72	1	02.53	1	01.72
	2	52.62	2	71.62	2	49.90
	3.1	45.66	3.2	25.86	4	48.38 (87.58,12.42)*
